# Case Report: Unusual High-Grade Diffuse Leptomeningeal Glioneuronal Tumor Mimicking Tuberculous Meningitis in a Child From an Endemic Region

**DOI:** 10.3389/fped.2021.767614

**Published:** 2021-12-09

**Authors:** Yong Guang Teh, Nornazirah Azizan, Nur Atifah Mohd Naim, Chiak Yot Ng, Ke Juin Wong, Faizah Mohd Zaki

**Affiliations:** ^1^Department of Radiology, Faculty of Medicine & Health Sciences, Universiti Malaysia Sabah, Kota Kinabalu, Malaysia; ^2^Department of Radiology, Sabah Women & Children's Hospital, Kota Kinabalu, Malaysia; ^3^Department of Pathology and Microbiology, Faculty of Medicine & Health Sciences, Universiti Malaysia Sabah, Kota Kinabalu, Malaysia; ^4^Department of Paediatrics, Sabah Women & Children's Hospital, Kota Kinabalu, Malaysia; ^5^Department of Radiology, National University of Malaysia Medical Centre, Kuala Lumpur, Malaysia

**Keywords:** diffuse leptomeningeal glioneuronal tumor, MR spectroscopy, imaging, tuberculosis, meningitis

## Abstract

**Background:** Diffuse leptomeningeal glioneuronal tumor (DL-GNT) is a new entity described in the 2016 World Health Organization (WHO) classification of brain tumors. While DL-GNT is predominantly an indolent tumor that affects young boys, high-grade DL-GNT is unusual and seldom reported in children.

**Case Presentation:** In this report, we describe the challenges and pitfalls associated with diagnosing this high-grade variant in a tuberculosis-endemic region. We highlight the importance of identifying non-typical imaging findings, i.e., non-enhancing cystic lesions with high T2 signal along the leptomeningeal surface, that may expedite the diagnosis of this condition. Histopathologic correlations with MR spectroscopy findings are also discussed.

**Conclusion:** We provide the first clinical imaging report of utilizing MR spectroscopy to distinguish DL-GNT from tuberculosis with histopathologic correlation.

## Introduction

Diffuse leptomeningeal glioneuronal tumor (DL-GNT) is a new entity described in the 2016 World Health Organization (WHO) classification of brain tumors (1). DL-GNT primarily affects male children, and the incidence in adults is exceedingly uncommon (1). Histologically, this rare entity is characterized by diffuse proliferation of oligodendroglial-like cells without infiltration to the brain parenchyma (2). Pediatric DL-GNT is typically an indolent tumor with a low proliferation rate, quantified by Ki-67 immunohistochemistry (2). In low-grade DL-GNT, a mean survival of 22 months has been documented, and 63% of patients die within 9 months after diagnosis (3). On the other hand, high-grade DL-GNT in children is rare and alludes to a more aggressive clinical course. At present, we do not know the precise incidence of this disease, because most of our knowledge is derived from isolated case reports and small case series.

The diagnosis of DL-GNT is challenging, as patients present with non-specific symptoms of raised intracranial pressure and focal neurology. Additionally, cerebrospinal fluid analysis is often misleading with patients having elevated protein and normal glucose levels, which is compatible with tuberculous meningitis. The difficult task of distinguishing this tumor from a granulomatous process is further compounded when DL-GNT presents in regions where tuberculosis (TB) is endemic.

A characteristic imaging finding of DL-GNT is diffuse enhancing nodular thickening of the meninges at the basal cisterns (4). Cystic leptomeningeal lesions, restricted diffusion, and cranial nerve palsies have been reported inconsistently in the extant literature (2). MR spectroscopy is a potentially useful sequence that can assist clinicians in distinguishing neoplastic growth from an underlying inflammatory process. Observation of a singlet peak at 3.8 ppm on MR spectroscopy suggests the diagnosis of TB meningitis (5). However, spectroscopy findings for high-grade DL-GNT have yet to be reported in the literature.

In this report, we describe the clinical presentation, laboratory results, imaging characteristics, and histopathologic findings of DL-GNT in a patient from a TB-endemic region.

## Case Report

A 5-year-old boy initially presented with headache, right eye pain, and vomiting, to a rural district hospital in the State of Sabah, East Malaysia on Borneo island. The boy also experienced difficulty in walking, blurring of vision, and fluctuating consciousness 1 month before admission. There was a significant (but unquantified) loss of weight and anorexia. He completed his bacille Calmette-Guerin (BCG) vaccination but missed all scheduled shots after his first birthday because of poor family support. On examination, a Glasgow Coma Scale (GCS) of 9 was documented (E2V2M5) with left lateral strabismus. The pupils were unequal (4 mm/3 mm) and sluggish. Power in all four limbs was 4/5 (Medical Research Council scale) with normal reflexes. Babinski was downgoing, and Kernig's sign was not elicited.

The boy was transferred to our center for further evaluation and treatment. Non-contrast CT brain showed acute hydrocephalus with cerebral edema. Subsequently, an external ventricular drain was inserted. Intra-operatively, the opening pressure was high, and outflowing cerebrospinal fluid (CSF) was clear and colorless.

**Figure 1:**
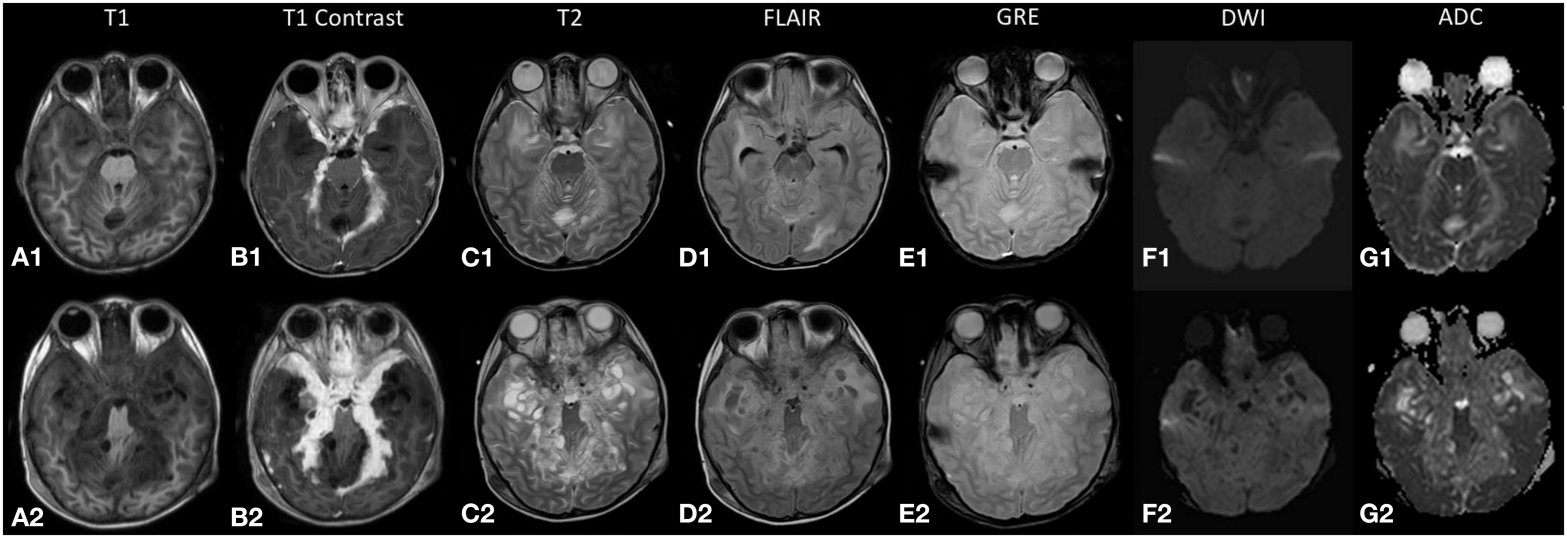
The top row **(A1–G1)** displays magnetic resonance (MR) images from the initial study during the first admission, and the bottom row **(A2–G2)** displays the corresponding images during the relapse 2 months later. The lesion **(A1,A2)** returns an isointense signal on T1-weighted images and **(B1,B2)** demonstrates enhancement on post-contrast images. **(C2)** High T2-signal cystic lesions are seen along the leptomeningeal surface that increased in size and number during the relapse. **(D2)** The internal signal of these cystic lesions is not entirely suppressed on fluid attenuation inversion recovery (FLAIR) sequence. **(E1,E2)** No blooming artifacts seen on gradient echo (GRE) sequence. **(F1,G1;F2,G2)** Restricted diffusion is not demonstrated in both studies.

**Figure 2:**
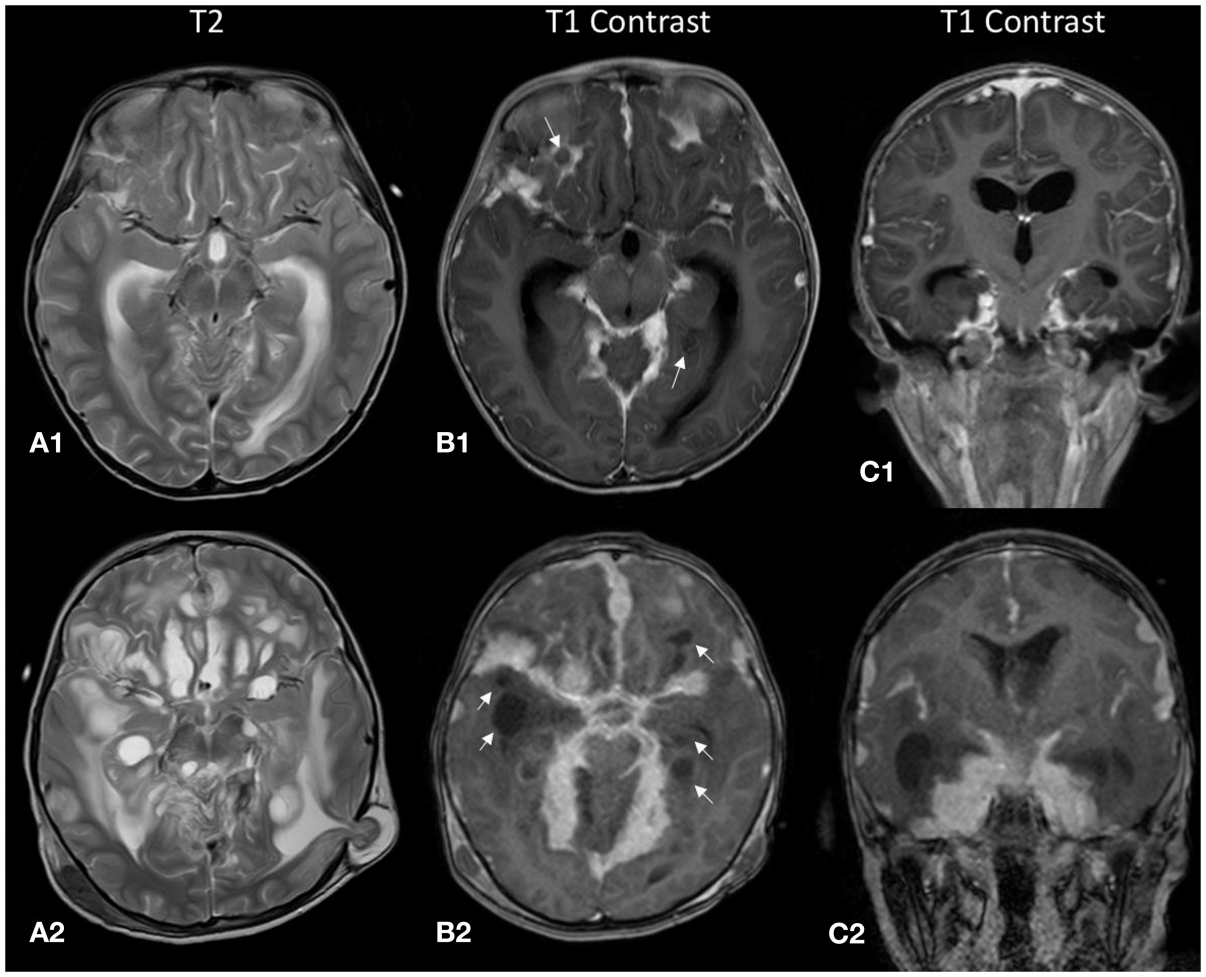
The top row **(A1–C1)** displays MR images from the initial study during the first admission, and the bottom row **(A2–C2)** displays the corresponding images during the relapse 2 months later. **(A1,A2)** There were cystic lesions with high T2 signal along the leptomeningeal surface. These initially small non-enhancing cystic lesions [white arrows, **(B1)**] increased in size and number in the follow-up scan [white arrows, **(B2)**]. **(A2)** Brain tissue was seen herniating through a Burr hole from a previous external ventricular shunt site, indicating that there was increased intracranial pressure. **(C1,C2)** Coronal post-contrast T1 images demonstrate the predominantly basal cisternal location of the nodular leptomeningeal thickening.

Magnetic resonance imaging of the brain revealed diffuse enhancing nodular leptomeningeal thickening, especially at the basal cisterns (Figure 1). No intra-axial lesion was present. Small non-enhancing cystic lesions were seen along the leptomeningeal surface (Figure 2B1), and no restricted diffusion was depicted. A diagnosis of tuberculous meningitis was considered, and an extensive TB workup was undertaken. The positive results from that battery of tests were a high erythrocyte sedimentation rate (ESR) of 90 mm/h and elevated CSF protein with normal CSF glucose levels. Otherwise, the Mantoux test was negative, and the blood, CSF, and CSF TB cultures showed no organism. The CSF for acid-fast bacilli as well as CSF GeneXpert were also negative. CSF latex agglutination was negative for *streptococcus* Group B, *haemophilus influenzae, streptococcus pneumoniae, neisseria meningitidis*, and *escherichia coli*. Both gastric lavage for acid fast bacilli stain and gastric lavage for GeneXpert were negative.

Smear-negative TB was considered after reviewing the biochemical and imaging findings. Using the Composite Reference Standard (CRS) criteria, this child was classified as *probable TB* (6). A standard anti-TB regimen was started consisting of isoniazid, rifampicin, pyrazinamide, and ethionamide. Intravenous dexamethasone was prescribed during the first 2 weeks of admission and was subsequently changed to oral administration.

The boy's condition improved considerably after 14 days, and he was well enough to undergo a comprehensive ophthalmology examination. He had a visual acuity of 1/60 in the right eye, and there was no light perception in the left eye. A positive right afferent pupillary defect was detected. Ophthalmoscopy showed normal macula bilaterally.

Both his parents were screened and found to be negative for TB. He was discharged well after a 4-week hospital stay.

**Figure 3:**
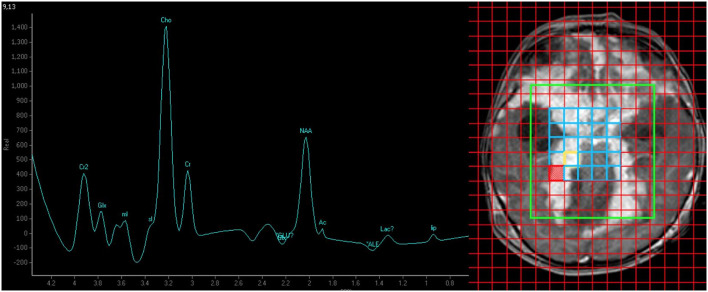
MR spectroscopy (TE/TR = 144 ms/2,000 ms) findings of elevated choline:creatine ratio (4.34) suggesting high cell turnover in favor of malignancy. The absence of a singlet peak at 3.8 ppm makes tuberculoma less likely.

One month later, he presented again with vomiting, unsteady gait, and anorexia. The follow-up MR brain study revealed exuberant nodular leptomeningeal enhancement that was more extensive than the initial study (Figure 1). Also, the previously seen cystic lesions have increased in size and number (Figures 2B2,C). MR spectroscopy (single voxel placed at the thickened tentorium cerebelli) showed a markedly elevated choline:creatine ratio of 4.34 and absence of a singlet peak at 3.8 ppm (Figure 3). A biopsy sample was obtained and sent for histopathological examination (HPE). Transcranial approach at the right Keen's point was used to obtain the biopsy sample. The patient incidentally needed a revision of his external ventricular shunt at that time, so dural tissue was obtained *via* the Burr hole just prior to the shunt revision.

**Figure 4:**
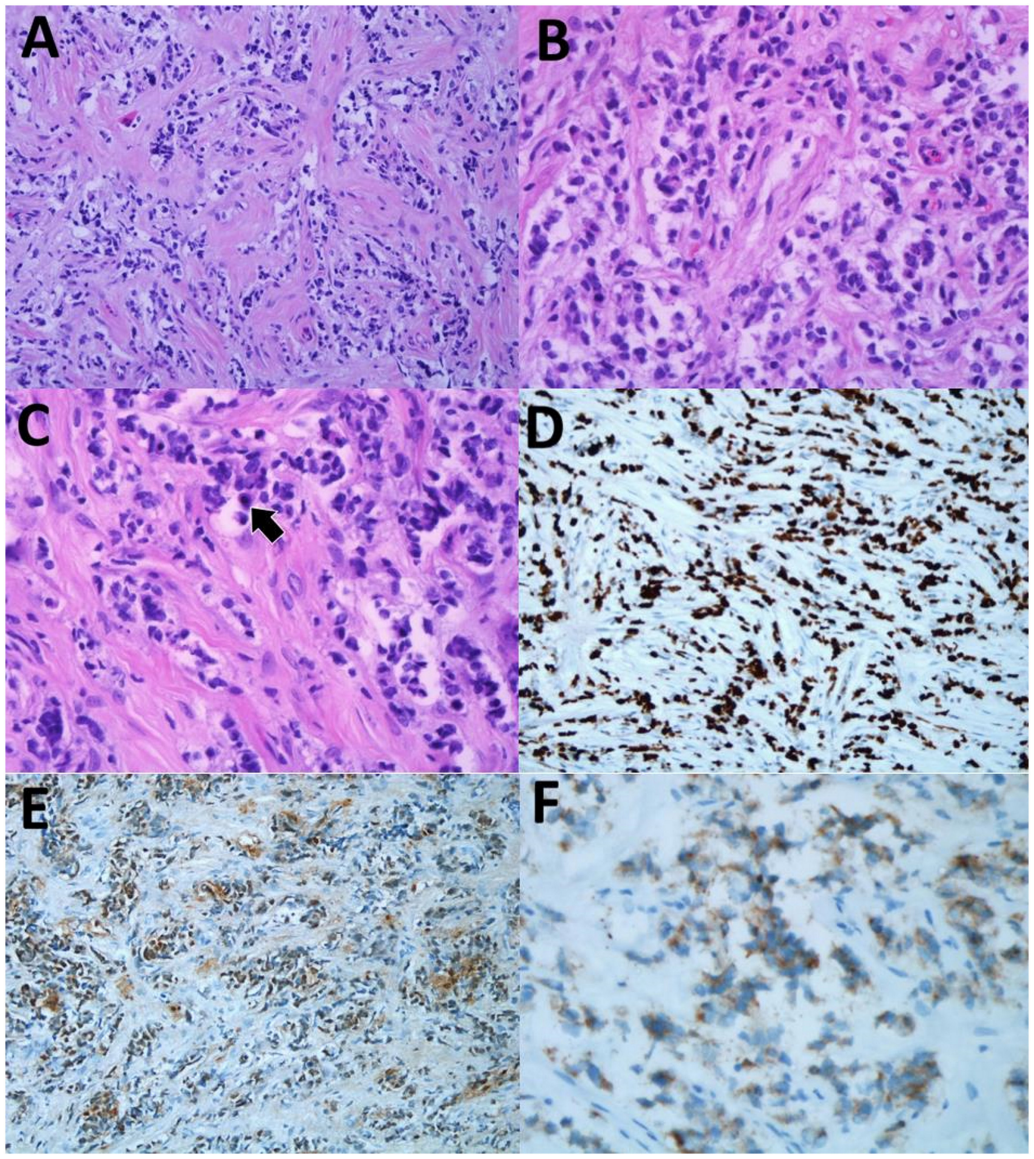
**(A)** The lesion showed moderate neoplastic cells with background desmoplastic and myxoid changes. (H&E, original magnification ×20). **(B)** The cells were fairly monomorphic with mild nuclear pleomorphism displaying enlarged round to oval nuclei with fine nuclear chromatin and inconspicuous nucleoli hematoxylin and eosin (H&E), original magnification ×40). **(C)** Occasional mitosis was seen (arrow) (H&E, original magnification ×40). **(D)** High Ki-67 proliferation rate of the neoplastic cells (immunohistochemical (IHC) Ki-67, original magnification ×20). **(E)** The neoplastic cells were positive for S100 (IHC S100, original magnification ×20). **(F)** The cells showed positivity toward synaptophysin immunostain (IHC synaptophysin, original magnification ×40).

Histopathological examination (HPE) revealed a moderately cellular neoplastic proliferation with background desmoplastic and myxoid changes seen from the hematoxylin and eosin (H&E) stain slides (Figure 4A). The cells were fairly monomorphic with mild nuclear pleomorphism displaying enlarged round to oval nuclei with fine chromatin and inconspicuous nucleoli (Figure 4B). Occasional mitosis was seen (Figure 4C). The Ki-67 proliferation rate was high, accounting for about 80% (Figure 4D). Immunohistochemical (IHC) studies showed that the tumor cells were positive for S100 and synaptophysin (Figures 4E,F). Given these findings, the boy was diagnosed with DL-GNT.

A family conference was held to counsel the parents on possible treatment options. The parents decided to opt for palliative treatment. The patient succumbed to the illness 3 months later, 22 weeks in total, after diagnosis.

## Discussion

Diagnosing DL-GNT in our context was challenging for two reasons. First, TB is endemic in our region, and many patients present late with multiple complications such as tuberculous meningitis (7). Based on the CRS, we classified our patient as *probable TB* given the clinical symptoms, imaging findings, and other supporting laboratory investigation results (6). Notwithstanding the smear-negative status of our patient, the nonspecific presenting symptoms, elevated ESR, suggestive CSF biochemistry, and initial imaging findings were all compatible with tuberculous meningitis (8). Second, the initial response to the intravenous dexamethasone and anti-TB agents served to strengthen our conviction that we were dealing with TB at that time. However, in hindsight, the administration of a potent steroid most likely caused temporary shrinkage of the tumor, which later rebounded when the drug was changed from intravenous to oral administration. Another consideration for the improved symptoms is the role of the external ventricular shunt in alleviating the increased intracranial pressure. The nonspecific visual deficits can be accounted for by pressure and mass effect from the rapidly growing tumor.

Locally, we routinely manage and treat culture-negative tuberculosis, and patients improve with empirical treatment. The presence of culture-negative tuberculosis is a perennial problem in our region (9). While the GeneXpert results were negative, we had to maintain a high degree of suspicion as we have local evidence to indicate that a small percentage of culture-negative TB patients can still be missed with GeneXpert testing (10). In the absence of a positive culture, we used the Composite Reference Standard (CRS) criteria to decide on whether a suspected patient should receive treatment for extrapulmonary tuberculosis (6). In endemic areas, the CRS is successful in helping pediatricians and physicians identify culture-negative tuberculosis (11). We did not perform a biopsy when the initial infective work-up was negative, because we were treating the patient as culture-negative extrapulmonary tuberculosis.

Retrospectively, familiarity with less common imaging findings of DL-GNT would have helped us reach a diagnosis earlier. The main imaging findings in our case were diffuse nodular leptomeningeal thickening and non-enhancing cystic lesions appearing along the leptomeningeal surface (Figures 2B1,B2). Initially, these lesions were subtle (Figure 2B1) but subsequently progressed to larger coalescing lesions predominantly at the basal cisterns (Figures 2A2,B2). These cystic lesions have been reported in only 29% of patients in an international case series (4). Also noteworthy was that, unlike another reported case of high-grade DL-GNT, the MR images in our study did not show impeded diffusion (2).

The MR spectroscopy findings helped us diagnose the high-grade DL-GNT and distinguish it from a chronic granulomatous process. The absence of a singlet peak at 3.8 ppm favored a malignancy over TB. This observation is based on the hypothesis that the singlet peak at 3.8 ppm represents guanidinoacetate (Gua), a molecule that is usually not present in high-grade tumors (5). Also, the markedly elevated choline:creatine ratio corresponded to the presence of nuclear pleomorphism and mitotic figures with a high Ki-67 proliferation rate and should be equally stressed as this points toward a malignant process. The aggressive nature of the disease led to the relatively early death (2). To the best of our knowledge, this is the first report on performing MR spectroscopy to aid in the diagnosis of DL-GNT.

Histologically, three criteria need to be met to diagnose DL-GNT: (1) glial cell proliferation without extension into the brain parenchyma, (2) absence of intra-axial lesions, and (3) demonstrable leptomeningeal encapsulation. In our case, leptomeningeal encapsulation could not be demonstrated, as the biopsy did not include tumoral tissue from the periphery. Nevertheless, the patient was diagnosed with DL-GNT, because the lesion was essentially within the leptomeningeal region with no primary brain parenchymal lesion. Furthermore, the positive immunohistochemistry S100 and synaptophysin staining are specific for DL-GNT (3). Based on our experience, we recommend that future biopsies should ideally include the peripheral tissue to enable the demonstration of the capsule.

There is currently no standardized treatment protocol for DL-GNT owing to the rarity of the disease. The mainstay of treatment includes a combination of chemotherapy and radiotherapy with varying degree of success (12). The most widely used chemotherapy agents are the carboplatin/vincristine combination and temozolomide. Chemotherapy appears to be able to halt the progression of the disease (13), while radiotherapy has been used to manage disease progression (12). One promising report documented complete remission with a combination of temozolomide and radiotherapy in a teenage patient (14). While the average survival period has been reported to be 22 months (3), more recent data indicate that the progression-free period may be more than 13 years (13).

## Conclusion

We have described the challenges and pitfalls associated with diagnosing a high-grade variant of DL-GNT in a tuberculosis-endemic region. We also highlighted the importance of identifying salient morphological and MR spectroscopy findings to aid the diagnostic process. While definitive treatment is still unavailable, high clinical suspicion and prompt recognition of salient imaging characteristics can help reduce unnecessary delays in reaching a firm diagnosis to enable further management planning.

## Data Availability Statement

The datasets presented in this article are not readily available because they contain identifiable protected health information. Requests to access the datasets should be directed to Yong Guang Teh (tehyongguang@ums.edu.my).

## Ethics Statement

Ethical review and approval was not required for the study on human participants in accordance with the local legislation and institutional requirements. Written informed consent to participate in this study was provided by the participants' legal guardian/next of kin.

## Author Contributions

YGT, NA, and NAMN wrote the initial draft manuscript. KJW, NA, CYN, and FMZ collected, reviewed, and verified the clinical, imaging, and histopathological data. All authors were involved in reviewing the manuscript.

## Funding

Article Publishing Charge (APC) was funded by Universiti Malaysia Sabah.

## Conflict of Interest

The authors declare that the research was conducted in the absence of any commercial or financial relationships that could be construed as a potential conflict of interest.

## Publisher's Note

All claims expressed in this article are solely those of the authors and do not necessarily represent those of their affiliated organizations, or those of the publisher, the editors and the reviewers. Any product that may be evaluated in this article, or claim that may be made by its manufacturer, is not guaranteed or endorsed by the publisher.

## Abbreviations

DL-GNT: diffuse leptomeningealglioneuronaltumor
HPE: histopathological examination
MR: magnetic resonance
TB: tuberculosis
WHO: World Health Organization.
